# The Impact of Autophagy on Cell Death Modalities

**DOI:** 10.1155/2014/502676

**Published:** 2014-02-04

**Authors:** Stefan W. Ryter, Kenji Mizumura, Augustine M. K. Choi

**Affiliations:** ^1^Pulmonary and Critical Care Medicine, Brigham and Women's Hospital, Harvard Medical School, 75 Francis Street, Boston, MA 02115, USA; ^2^Weil Cornell Medical College, 525 East 68th Street, Room M-522, Box 130, New York, NY 10065, USA

## Abstract

Autophagy represents a homeostatic cellular mechanism for the turnover of organelles and proteins, through a lysosome-dependent degradation pathway. During starvation, autophagy facilitates cell survival through the recycling of metabolic precursors. Additionally, autophagy can modulate other vital processes such as programmed cell death (e.g., apoptosis), inflammation, and adaptive immune mechanisms and thereby influence disease pathogenesis. Selective pathways can target distinct cargoes (e.g., mitochondria and proteins) for autophagic degradation. At present, the causal relationship between autophagy and various forms of regulated or nonregulated cell death remains unclear. Autophagy can occur in association with necrosis-like cell death triggered by caspase inhibition. Autophagy and apoptosis have been shown to be coincident or antagonistic, depending on experimental context, and share cross-talk between signal transduction elements. Autophagy may modulate the outcome of other regulated forms of cell death such as necroptosis. Recent advances suggest that autophagy can dampen inflammatory responses, including inflammasome-dependent caspase-1 activation and maturation of proinflammatory cytokines. Autophagy may also act as regulator of caspase-1 dependent cell death (pyroptosis). Strategies aimed at modulating autophagy may lead to therapeutic interventions for diseases in which apoptosis or other forms of regulated cell death may play a cardinal role.

## 1. Introduction

**Figure 1:**
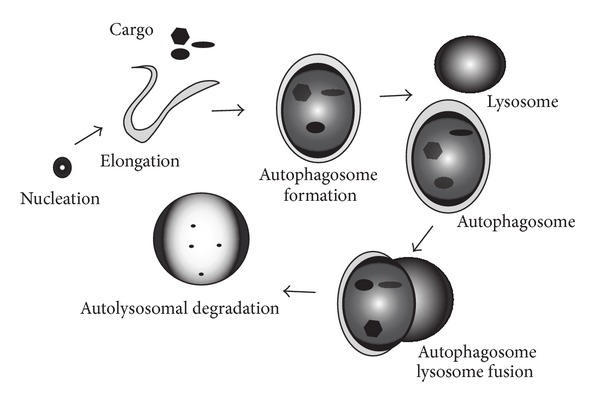
Autophagy pathway. Autophagy is a membrane-dependent pathway that involves a defined series of steps. The pathway is initiated by the autophagosome nucleation step (formation of a preautophagosomal structure leading to an isolation membrane or phagophore). This is followed by autophagosome membrane elongation. The next steps involve the formation of the mature autophagosome, which engulphs cytosol or specific substrates such as mitochondria or ubiquitinated proteins. Subsequently, the autophagosome containing its cargo fuses with the lysosome. In the autolysosome, the autophagosomal cargoes are digested by lysosomal hydrolases and the contents released for metabolic recycling.

Macroautophagy (abbreviated as “autophagy”) is a genetically regulated and evolutionarily conserved pathway for the degradation of subcellular components [1–5]. This process involves the *de novo* formation of cytoplasmic double membrane-bound vacuoles termed autophagosomes, which sequester cytosolic cargo for delivery to the lysosomes [5, 6]. Autophagic cargoes may include various subcellular targets typified by ubiquitin-modified or long-lived proteins and major cytosolic organelles (e.g., mitochondria and peroxisomes) [7–9]. However, a number of other potential substrates have been identified, including lipids, nucleic acids, reticulocytes, and invading pathogens (e.g., intracellular bacteria, viruses, etc.) [7, 10]. The autophagic pathway proceeds through several defined steps: (i) the initiation phase involving the formation of an isolation membrane or phagophore, (ii) the elongation of the phagophore, (iii) the maturation of an autophagosome with assimilation of a cytosolic cargo, (iv) the fusion of the mature autophagosome to the lysosome, and finally (v) the degradation phase where the contents are digested by lysosomal proteases (e.g., cathepsins) and other hydrolytic enzymes [1–5] (Figure 1). Autophagy has been recognized as an essential function for cell homeostasis and adaptation to environmental stress conditions including nutritional starvation, energy depletion, endoplasmic reticulum stress, oxidative stress, and hypoxia [11–14]. Furthermore, autophagy plays a vital role in innate and adaptive immune mechanisms, including resistance to pathogen infections [10, 15, 16]. The role of autophagy in diseases is an emerging area of investigation, with recent studies indicating that autophagy may exert multifunctional roles in specific diseases, with the potential for both adaptive and maladaptive outcomes. Furthermore, deficiency or absence in autophagic function may also contribute to the pathogenesis of human diseases [2, 12, 17–19].

The occurrence of autophagy in response to environmental stress, most notably starvation, is generally regarded as a cell survival mechanism [20–22]. Due to the often coincident appearance of morphological and biochemical markers of autophagy in cells that are dying, the relationship between autophagy and cell death has been both extensively studied and speculated upon [23–26]. Autophagy has previously been classified as a form of programmed cell death, termed “autophagic cell death” to describe a form of caspase-independent necrosis-like cell death associated with accumulation of autophagosomes in cells [27]. This classification is now controversial, and the casual relationship between autophagy and cell death remains unproven [25, 26]. Nevertheless, many studies have pointed to intimate relationships between autophagy and cellular death programs, which are not yet fully understood [28]. Recent studies have also examined potential cross-talk between the signaling pathways that regulate autophagy and those that regulate distinct forms of regulated cell death such as apoptosis [29]. Current advances in these areas will be summarized in this review.

### 1.1. Modes of Cell Death

The major types of cell death which have been studied most extensively in the context of autophagy research include apoptosis, necrosis, necroptosis, and pyroptosis, as briefly summarized here.

#### 1.1.1. Apoptosis

Apoptosis denotes a regulated form of cell death that requires the coordinated action of proteases and nucleases within an intact plasma membrane. Morphological characteristics of apoptosis include DNA fragmentation, plasma membrane blebbing, cell shrinkage, and cellular decomposition into membrane-bound apoptotic bodies which are removed by phagocytosis [30–33]. The cardinal biochemical features of apoptosis include mitochondrial dysfunction, respiratory chain inhibition, loss of inner mitochondrial membrane potential (Δ*Ψ*_*m*_), increased mitochondrial membrane permeability, and externalization of phosphatidylserine [30–33]. Apoptosis has a crucial function in the maintenance of tissue homeostasis under physiological conditions and also serves as a component of developmental programs and furthermore may also contribute to disease pathogenesis. Several intracellular signaling pathways may activate apoptosis. The “intrinsic” (mitochondria-dependent) apoptotic pathway represents a major mechanism by which exposure to harmful extracellular stimuli triggers apoptosis. This pathway is dependent on a proteolytic activation cascade for both regulation and execution (i.e., caspases) and subject to regulation by Bcl-2 family proteins. The extrinsic apoptotic pathway, which shares common downstream features with the intrinsic pathway, is defined by its dependence on receptor-ligand (e.g., Fas-Fas ligand) interactions for initiation.

#### 1.1.2. Necrosis

Necrosis is a type of cell death that results from acute, accidental, or nonphysiological injury [30–33]. This type of cell death is associated with cell lysis as the consequence of membrane damage and subsequent leakage of cell constituents into the extracellular space, which may lead to local inflammation and damage to the surrounding tissue. In certain cases, cell swelling or oncosis may precede necrosis [33]. Necrosis and apoptosis differ in morphological features, though the two processes are not necessarily mutually exclusive. Both apoptosis and necrosis can occur in response to treatment with many injurious stimuli, usually in a dose-dependent fashion. Many agents that cause apoptosis at low to moderate doses may ultimately cause necrosis at relatively higher doses. A number of endogenous events can determine the balance between apoptotic and necrotic death. Cellular energy charge (i.e., ATP levels) may represent one such factor that influences cell fate decisions. Whereas ATP is required for certain steps of caspase activation, rapid decline of cellular ATP levels typically leads to necrotic cell death.

#### 1.1.3. Necroptosis

The existence of necrotic cell death pathways regulated by an intrinsic death program distinct from that of apoptosis has also been proposed. A regulated Fas-dependent but caspase-independent nonapoptotic cell death, termed “*necroptosis*,” that resembles necrosis has been described [34, 35]. In this form of regulated necrosis, the ligand binding of death receptors such as the tumor necrosis factor receptor 1 (TNFR1) can promote the formation of a macromolecular complex (necrosome), involving the receptor-interacting protein (RIP) kinase-1 and kinase-3 that initiate necrosis [36, 37]. Increasing evidence affirms the relevance of this mode of cell death in the pathogenesis of various diseases [38–42].

#### 1.1.4. Pyroptosis

Pyroptosis represents a form of cell death that is triggered by proinflammatory signals and which is associated with inflammation [32, 43, 44]. This type of cell death occurs primarily in inflammatory cells such as macrophages and may be triggered by bacterial or pathogen infections. A major feature of pyroptosis is the requirement for caspase-1 activation. Caspase-1 is responsible for the maturation of proinflammatory cytokines such as IL-1*β* and IL-18 through inflammasome-dependent pathways. Cells undergoing pyroptosis release increased amounts of IL-1*β* and IL-18. The execution of pyroptosis may also require caspase-7. Cells undergoing pyroptosis share some common features of necrosis. Cell death occurs as a result of membranous pore formation and cytoplasmic swelling and leakage of cytosolic contents. Similar to apoptotic cells, pyroptotic cells may also display DNA fragmentation and nuclear condensation.

### 1.2. Molecular Regulation of Autophagy

The autophagic pathway is highly regulated by a genetic program. The molecular machinery of autophagic regulation is the subject of recent reviews [45, 46]. Subsequent to their identification in yeast, a number of critical autophagy-related genes (Atg) have been identified whose gene products regulate distinct steps in the induction or progression of autophagy [45, 46].

In brief, the autophagy pathway responds to regulation by nutrient status, including nutrient deficiency (starvation) and loss of energy charge [47]. Starvation induces autophagy through the inhibition of mammalian target of rapamycin (mTOR), which resides in a multiprotein complex, mTORC1 [47]. In response to stimulation by nutrients or growth factors, mTORC1 negatively regulates a macromolecular substrate complex that includes ULK1, ATG13, ATG101, and FIP200 (RB1CC1), which results in autophagy suppression [48–54]. Energy depletion, which stimulates autophagy, inhibits mTORC1, in part through activation of the AMP-dependent protein kinase (AMPK), leading to the activation of ULK1, an important initiating step in autophagy [47, 55, 56].

Autophagy is also coregulated by a multiprotein complex consisting of Beclin 1 (homologue of yeast Atg6), which associates with class III phosphatidylinositol-3-kinase (VPS34) and a number of additional stimulatory or inhibitory coregulatory proteins (e.g., ATG14L, UVRAG, Ambra1, and Rubicon) [57]. In response to proautophagic stimuli, the increased production of phosphatidylinositol-3-phosphate (PI3P) by this complex regulates autophagosome formation [57, 58]. The Beclin 1 complex is subject to negative regulation by the PI3 K/Akt pathway [59] as well by binding interactions with antiapoptotic Bcl-2 family proteins [60]. Following phagophore formation, the elongation of the autophagosome membrane requires the action of two ubiquitin-like conjugation systems: the Atg5-Atg12 conjugation system and the microtubule-associated protein-1 light chain 3 (LC3, Atg8) conjugation system [61, 62]. Atg4B converts the proform of LC3B to its cytosolic free form (LC3-I). In mammals, the conversion of LC3-I (and other Atg8 homologues) to its phosphatidylethanolamine-conjugated and autophagosome-membrane associated form (i.e., LC3-II) is an initiating step in autophagy [63–66].

## 2. Autophagy in Cellular Homeostasis

Autophagy is now recognized to play multifunctional roles in the maintenance of cellular homeostasis. Once thought to be relatively nonspecific, it is now believed that autophagy is a highly selective process in which distinct cellular mechanisms are employed to identify and target cargo to autophagosomes. Such selective autophagy pathways have been identified for the turnover of mitochondria (mitophagy) and other organelles and the turnover of denatured protein (aggrephagy).

### 2.1. Cell Survival during Starvation

During starvation (e.g., deprivation of glucose or growth factors or depletion of cellular energy charge) autophagy prolongs cell survival through the degradation and recycling of cellular macromolecules. This process replenishes pools of precursor molecules during nutrient deficiency states [20]. Mice deficient in the autophagy protein Atg5 are susceptible to the lethal effects of starvation [21]. Inhibition of autophagy by Beclin 1 or Atg5 knockdown, or by chemical inhibitors such as 3-methyladenine, can promote apoptosis and caspase-3 activation in starved HeLa cells [22]. These studies have suggested a role for autophagy as a means for prolonging cell survival during starvation.

### 2.2. Mitophagy

Autophagy performs a cardinal homeostatic function in the removal of damaged or dysfunctional mitochondria, in a selective process referred to as mitophagy [9]. Mitophagy plays an important role in erythrocyte maturation and the maintenance of cellular homeostasis. The increased turnover of mitochondria by mitophagy may occur as a result of chemical or physical stress (e.g., hypoxia) [67]. Mitophagy can regulate mitochondrial number to match metabolic requirements. Mitochondria are removed during erythrocyte maturation by the BH3-only protein, Nix/Bnip3L1. Nix localizes in the outer mitochondrial membrane and directly interacts with mammalian Atg8 homologs through its LIR motif [68]. Damaged or dysfunctional mitochondria are recruited to the autophagosome for removal by mitophagy through a process regulated by the phosphatase and tensin homolog deleted in chromosome 10 (PTEN)-induced putative kinase 1 (Pink1) and Parkinson protein-2 (Parkin) [9, 69, 70]. Mutations in the corresponding *PINK1* and *PARK2* genes are associated with recessive familial forms of Parkinson's disease [71]. In mice, *PINK1* and *PARK2* deletions are associated with mitochondrial dysfunction [72]. Loss of mitochondrial membrane potential and the increased production of mitochondrial reactive oxygen species (ROS) may provide initiating signals for mitophagy. Pink1, a transmembrane protein, is stabilized on damaged or depolarized mitochondria. Following the decline of mitochondrial membrane potential, which can be caused by chemical stress, Pink1 recruits cytosolic Parkin, an E3 ubiquitin protein ligase, to the mitochondria [69, 70, 73]. Parkin initiates the formation of polyubiquitin chains which identify depolarized mitochondria for degradation. Parkin ubiquitinates mitochondrial outer membrane proteins including porin, mitofusin, and Miro [74, 75]. Ubiquitinated mitochondria are subsequently recognized and targeted to autophagosomes by the autophagic cargo adaptor protein p62 [9, 69, 70].

### 2.3. Aggrephagy in the Maintenance of Proteostasis

Autophagy can maintain cellular protein homeostasis (proteostasis) by providing a mechanism for the removal of ubiquitinated protein aggregates, in a selective process termed *aggrephagy* [8]. Recent studies suggest that autophagy may provide an alternative pathway to proteolysis in addition to the ubiquitin proteasome system [76–78]. Aggrephagy requires the selective autophagy cargo adaptor p62/SQSTM1 (p62) which can interact with ubiquitinated proteins through a ubiquitin-associated (UBA) domain [76]. Furthermore, p62 can interact with LC3 through its LIR (LC3-interacting region) motif and thereby facilitate the targeting of ubiquitinated proteins to autophagosomes [77]. The selective autophagy adaptor, NBR1 (neighbor of BRCA1 gene 1), promotes the formation of ubiquitin-positive protein aggregates, facilitating their sequestration and removal by aggrephagy [78]. This process involves the 400 kDa, PI3P-binding autophagy-linked FYVE domain protein (ALFY), a p62-interacting protein [79].

### 2.4. Other Forms of Selective Autophagy

In addition to mitophagy, other forms of organelle-specific or substrate-specific autophagy have been identified and collectively may contribute to the maintenance of cellular integrity under stress. These include the selective autophagic degradation of peroxisomes [80], ribosomes [81], and endoplasmic reticulum fragments [82]. In addition to protein, autophagic processes have been implicated in the degradation of diverse cellular biomolecules, including lipids [83] and RNA [84]. Furthermore, autophagy can degrade exogenously derived substrates, most notably bacteria, virus particles, and other parasites, in a selective process termed “xenophagy” [10, 15, 16]. Although recent studies begin to unravel the role of mitochondrial selective autophagy in cell death pathways, the role of diverse selective autophagy pathways in the modulation of cell death programs remains largely uncharted territory.

## 3. Autophagy and Apoptosis

Despite a widely accepted role for autophagy in cellular survival, autophagy has also been associated with the regulation of various cell death pathways, most notably apoptosis. Autophagy is a regulated program associated with survival or stress adaptation. However, increased autophagosome formation is often coincident in cells that are dying. Thus, autophagy may represent a failed adaptive mechanism that may have prevented death under milder conditions. Hypothetically, excess activation of autophagy may contribute to apoptotic cell death through unchecked degradative processes [23]. The morphological and biochemical features of autophagy and apoptosis are distinct. Cells undergoing autophagy display an increase in autophagic vesicles (i.e., autophagosomes and autophagolysosomes). While partial chromatin condensation appears in autophagic cells, DNA fragmentation does not occur. The distinctions between autophagy and apoptosis remain incompletely delineated, as the two processes are not always mutually exclusive and may occur simultaneously in the same cell type.

### 3.1. Cross-Talk between Autophagy and Apoptosis Proteins

**Figure 2:**
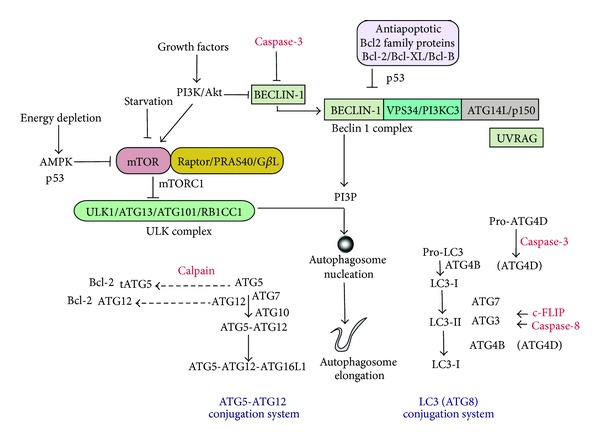
Autophagy is negatively regulated by the phosphatidylinositol 3-kinase (PI3 K)/Akt signaling pathway, which activates mammalian target of rapamycin (mTOR) in response to growth factors and also phosphorylates Beclin 1. The adenosine 5′-monophosphate-activated protein kinase (AMPK) negatively regulates mTOR thereby acting as a positive regulator of autophagy in response to AMP levels. mTOR resides in the mTOR signaling complex (mTORC1), which regulates the mammalian uncoordinated-51-like protein kinase (ULK1) complex, consisting of ULK1, ATG13, ATG101, and RB1CC1. Autophagy is also regulated by the Beclin 1 complex, consisting of Beclin 1, class III phosphatidylinositol-3-kinase (VPS34 or PI3KC3) and ATG14L or UVRAG. Stimulation of the Beclin 1 complex generates phosphatidylinositol-3-phosphate (PI3P), which triggers autophagosomal nucleation. Autophagosome membrane elongation is regulated by ubiquitin-like conjugation systems. ATG12 is conjugated to ATG5 by ATG7 and ATG10 enzymes, which results in the formation of the ATG5-ATG12-ATG16L1 complex. The Atg8 conjugation system involves microtubule-associated protein-1 light chain 3 (LC3, ATG8). LC3 and other Atg8 homologues are modified with the cellular lipid phosphatidylethanolamine. Pro-LC3 is cleaved by ATG4B to generate the LC3-I form. ATG4D may similarly process other Atg8 homologs. Lipid conjugation of LC3-I occurs from the action of ATG7 and ATG3 activities. The conversion of LC3-I (free form) to LC3-II (lipid-conjugated form) is a major step in autophagosome formation. Antiapoptotic Bcl-2 family proteins can interact with the autophagy machinery at the site of Beclin1, resulting in inhibition of autophagy. The tumor suppressor protein p53 and activated caspases potentially interact with the autophagy machinery at the indicated sites. Caspase processing can result in activation of autophagy (e.g., Atg4D) or inhibition of autophagy (e.g., Beclin 1, Atg3). Additionally, Atg12 and a cleavage product of Atg5 can act as proapoptotic mediators by antagonizing antiapoptotic Bcl-2 family proteins.

Recent studies suggest that factors well known to regulate apoptosis pathways also have the potential to exert regulatory activity on factors that regulate autophagy and *vice-versa* (Figure 2). How these regulatory events, termed “cross-talk”, are integrated into a mechanism for the determination of cell fate yet remains incompletely understood.

Antiapoptotic Bcl-2 family proteins, which downregulate apoptosis (i.e., Bcl-2) by antagonizing the activity of proapoptotic proteins, can downregulate autophagy. Beclin 1 interacts with antiapoptotic Bcl-2 family members including Bcl-2 and Bcl-X_*L*_. Binding of these Bcl-2 family proteins to Beclin 1 inhibits autophagy by preventing the association of Beclin 1 with the class III PI3K complex [57, 60]. Recent studies have identified Bcl-B as a novel Beclin 1 binding protein [85]. BNIP3 is a BH3-only protein that can trigger apoptosis by sequestering antiapoptotic Bcl-2 family proteins and promoting Bax/Bad dependent mitochondrial release of proapoptotic mediators. BNIP3 also stimulates mitophagy by disrupting the interaction between Bcl-2 and Beclin 1 [86]. These interactions suggest that autophagy and apoptosis may be coordinately regulated by Bcl-2 family proteins. Experimental evidence also suggests that, once activated, apoptosis effector molecules may suppress autophagy; for example, Beclin 1 may be cleaved and inactivated by caspases during activation of apoptosis [87].

Further studies suggest that certain Atg proteins may play dual roles in autophagy/apoptosis regulation; for example, the autophagic protein Atg5 may affect extrinsic apoptosis pathways through interactions with the Fas-associated death domain (FADD) protein [88]. Atg5 which regulates autophagy can be subject to calpain-dependent cleavage to generate a proapoptotic truncation product (tAtg5). This cleavage product promotes apoptosis by binding to and inhibiting antiapoptotic proteins such as Bcl-X_*L*_ [89]. Recent studies also implicate Atg12, the binding partner for Atg5 required for autophagosomal elongation, as an effector of the intrinsic apoptosis pathway [90]. Atg12 may bind to and inactivate antiapoptotic Bcl2 family proteins (e.g., Bcl-2 and Mcl-1), through an interaction involving a BH2 motif, and thereby act as a proapoptotic regulator [90].

Recent advances have identified other regulatory targets for caspase regulation among autophagy related molecules; for example, Atg4D, a member of the Atg4 family of Atg8 processing enzymes, has been identified as a substrate for proapoptotic caspase-3 [91]. Caspase processing of Atg4D results in activation with respect to proautophagic activity. The autophagic protein Atg3 has recently been identified as a caspase-8 substrate, which is cleaved during TNF*α*-induced apoptosis [92]. The antiapoptotic protein cellular Flice-like inhibitory protein (c-FLIP) can act as a negative regulator of autophagy [93]. The c-FLIP, an endogenous inhibitor of caspase-8 processing and the extrinsic apoptotic pathway, acts to prevent the binding of Atg3 to LC3, which impairs LC3 processing [93].

On the basis of what is known about the molecular cross-talk between autophagy and apoptosis it currently remains unclear whether autophagy and apoptosis are coregulated or mutually exclusive processes. Antiapoptotic (e.g., Bcl-2) as well as proapoptotic (e.g., caspase-3) molecules can downregulate autophagy by interacting with Beclin 1. Furthermore, other caspase-dependent events have been implicated in anti-autophagy or proautophagy events. For example caspase processing of Atg4D is proautophagy, whereas caspase processing of Beclin 1 is antiautophagy.

The definitive cellular mechanisms that control the decision to embark on each one or both of these pathways in response to specific stimuli remain unclear. Analysis of any single isolated regulatory component (using siRNA knockdown for example) for its potential to cross-regulate autophagy and/or apoptosis will be unlikely to answer these questions. Thus, an integrative approach is needed to understand how the entire molecular machinery of apoptosis and autophagy are coordinated to influence cell fate decisions.

### 3.2. The Tumor Suppressor p53 Coregulates Autophagy and Apoptosis

The p53 tumor suppressor protein is a well studied regulator of cell cycle progression and apoptosis. p53 modulates the expression of Bcl-2 family proteins (e.g., Bax, Bid) and other apoptosis-related gene targets (e.g., Apaf1). The nuclear form of p53 targets the expression of DRAM (damage regulated autophagic modulator), which can stimulate both autophagy and apoptosis [94]. Alternatively, p53 can induce autophagy through the upregulation of AMPK, which downregulates the mTOR pathway [95]. Recent studies have shown that genetic or pharmacological inhibition of p53 can also activate autophagy and have led to the identification of the cytoplasmic form of p53 as an inhibitor of autophagy [96]. Chemical stimuli known to induce autophagy can promote the proteasomal degradation of p53 [96].

Cellular stimulation with interferon-*γ* (IFN-*γ*) induces the deacetylation of p53, leading to suppressed Bmf expression, reduced complex formation between Beclin 1 and Bcl2, and enhanced autophagy [97]. Taken together these studies suggest a complex role of p53 in the regulation of autophagy, with opposing roles for the cytosolic and nuclear forms of p53 [98, 99].

### 3.3. Autophagy as a Protagonist of Apoptosis

Several recent studies, supported by genetic manipulation of the autophagy program, have revealed that in select toxicological models, autophagy may be associated with the promotion of apoptosis.

In our recent studies we have found that epithelial cells subjected to cigarette smoke extract (CSE) exposure die by activation of the extrinsic apoptosis pathway [100, 101]. CSE-induced cell death involved activation of the Fas-dependent death-inducing signaling complex (DISC) and downstream activation of caspases (-8,-9,-3). Epithelial cells subjected to CSE exposure concurrently responded with increased autophagosome formation and increased processing of LC3B-I to LC3B-II in epithelial cells [100, 101]. Knockdown of autophagy proteins Beclin 1 or LC3B inhibited apoptosis in response to CSE exposure *in vitro*, suggesting that increased autophagy occurred in association with epithelial cell death [100, 101]. Further studies revealed that LC3B may act as a regulatory factor in extrinsic apoptosis in this model [101]. LC3B was found to engage a complex with Fas, the key component of the DISC, in a fashion dependent on the lipid raft protein caveolin-1. CSE exposure caused the rapid dissociation of LC3B from Fas, in association with the activation of apoptosis signaling [101]. In conclusion, these results using genetic knockdown experiments have implicated a proapoptotic role for LC3B, in a specialized model of CSE-induced toxicity, though the relative role of autophagic activity in promoting cell death in this model remains unclear [100, 101].

It should be noted that CSE-induced autophagy may differ from starvation-induced autophagy in that it occurs in the presence of a complex mixture of foreign matter, which may potentially alter the functionality of the autophagy response. Thus, the concept of “toxic autophagy” may involve altered function, which may be dependent not only on whether its activation is physiological or excessive, but also on the nature of foreign substrates (e.g., complex xenobiotics such as tar or virus particles) and their interactions with autophagosomes.

Further examples of coincident autophagy and apoptosis include p53-dependent autophagy through upregulation of DRAM, which is coincidental with upregulation of apoptosis [93]. TNF*α* can induce autophagy in trophoblasts leading to activation of the intrinsic apoptosis pathway [102]. Knockdown of Atg5 prevented TNF*α*-dependent activation of proapoptotic caspases in this model [102]. Deletion of Atg5 was also shown to protect cells from prodeath environmental stimuli; however, the authors attributed this resistance to compensatory activation of chaperone-dependent autophagy, rather than inhibition of macroautophagy *per se* [103].

These studies raise an important issue in that genetic knockdown of one specific autophagy-related factor cannot establish whether autophagy was protective or not in any context, as downregulation of the target may potentially affect signaling pathways that are independent of autophagy, or alternatively, promote compensatory mechanisms, such as alternate forms of autophagy.

## 4. Autophagy, Necrosis, and Necroptosis

### 4.1. Autophagy Dependent Cell Death during Apoptosis Inhibition

The terms “autophagic cell death” or type II programmed cell death have been previously used to refer to cell death distinct from apoptosis that occurs in association with increases in autophagosome formation and independently of caspases [26]. Many studies that have implicated autophagy as a cell death effector have been performed on apoptosis-compromised or caspase-deficient cells; for example, cells treated with z-VAD-*fmk*, a general inhibitor of caspases, or with caspase-8 and calpain inhibitors, die essentially by a nonapoptotic pathway characterized by dramatic accumulations of autophagic vacuoles [104–107]. Genetic knockdown experiments (e.g., *Beclin 1*) suggest that autophagy contributes to cytotoxicity in these models [104]; however, contrasting studies, also using knockdown of autophagy proteins, have also suggested that autophagy can also protect in the context of nonapoptotic cell death induced by caspase inhibition [108]. In *Bax* ^−/−^ *Bak* ^−/−^ mouse embryonic fibroblasts (MEFs), which cannot activate intrinsic apoptosis, treatment with chemotherapeutic agents results in nonapoptotic necrosis-like cell death accompanied by excessive autophagosome formation [109].

Currently it remains unclear whether the process of autophagy acts as an effector or bystander of caspase-independent necrosis-like cell death, though autophagic proteins likely play an accessory role [25, 26].

### 4.2. Cross-Talk of Autophagy and Necrosis

Experiments in tumor cells have suggested the possibility of cross-talk between autophagy and necrosis in cells [110]. Autophagy provides a protective function to limit tumor necrosis and inflammation in response to metabolic stress. While autophagy acts to buffer metabolic stress, the combined impairment of apoptosis and autophagy promotes necrotic cell death *in vitro* and *in vivo* [111]. Although it remains to be determined what triggers necrosis in tumor cells, it is likely that insufficient ATP production to maintain plasma-membrane integrity results in metabolic catastrophe and cell lysis [110, 112]. A rapid drop in ATP has been implicated in necrosis [113]. Autophagy integrates a metabolic feedback system to allow sufficient ATP generation to maintain cell viability [114]. Enhanced autophagy by spermidine, a natural polyamine, inhibits loss of membrane integrity and release of chromatin protein high mobility group B1 (HMGB1), a biomarker of necrosis [115].

Necrosis was once described as accidental cell death by extreme physicochemical stress. However, recent consensus agrees that specific genes can regulate necrosis, which is termed necroptosis [35]. The kinases receptor-interacting protein 1 (RIP1) and RIP3 are key signaling molecules in necroptosis. Published studies have suggested that the treatment with zVAD, a caspase inhibitor with broad specificity, induced autophagy and the death of L929 cells; and this death process required RIP1, suggesting that autophagy is involved in necroptosis [107]. In several models, autophagy has been shown to regulate necroptosis [116, 117]. In endothelial cells, inhibition of autophagy rescues palmitic acid-induced necroptosis [118]. On the other hand, a recent study has demonstrated that necrostatin-1 (Nec-1), a specific necroptosis inhibitor, suppressed not only necrosis but also autophagy [119]. These observations suggest that autophagy may be induced by necroptosis [120], raising the possibility that cellular stress during cell death may lead to the induction of autophagy. The molecular mechanism underlying this relationship remains elusive and controversial [121]. It is tempting to speculate that so-called autophagic cell death may involve elements of necroptosis, though further research will be needed to clarify this relationship, as well as the signaling pathways linking autophagy to necroptosis.

## 5. Autophagy, Inflammasome Activation, and Cross-Talk to Pyroptosis 

Recent observations have revealed a relationship between autophagic proteins and inflammasome-associated proinflammatory cytokine maturation in macrophages [122–124]. Inflammasomes are cytosolic multiprotein complexes that constitute a novel inflammatory signaling mechanism and which govern the maturation and secretion of distinct proinflammatory cytokines, such as IL-1*β*, IL-18, and IL-33 [125]. Cytosolic receptors of the Nod-like receptor (NLR) family (i.e., NLRP3, NLRP1) interact with accessory proteins to form inflammasome complexes. NLRP3 interacts with an adaptor protein [apoptosis-associated speck like protein containing CARD (ASC)], which recruits and activates the procaspase-1 by proteolytic cleavage [125].

Proinflammatory cytokine secretion (IL-1*β* and IL-18) was enhanced in *atg16l1* or *atg7* deleted macrophages in response to LPS [122]. In contrast, *atg16l1* or *atg7* deficiency did not affect TNF and IFN-*β* production or NF-*κ*B pathway activation in macrophages stimulated with LPS [122]. Furthermore, *atg16l1* deleted mice displayed increased susceptibility to a murine model of colitis, which could be ameliorated by anti-IL-18 therapy [122]. Increased activation of IL-1*β* and IL-18 has also been observed in macrophages and monocytes isolated from mice genetically deficient in Beclin 1 and LC3B [123]. Cytokine activation in response to LPS and ATP in wild-type macrophages, as well as the amplification observed in LC3B or Beclin 1 deficient macrophages, required the NLRP3 inflammasome pathway [123, 124]. The mechanism by which autophagy deficiency enhanced NLRP3 inflammasome pathway activation involved mitochondrial dysfunction, including the enhanced production of mitochondrial ROS and increased mitochondrial membrane permeability transition [122, 123]. The pathway to caspase-1 dependent IL-18 secretion in macrophages was inhibited by mitochondrial targeting antioxidants [123]. These experiments suggest that autophagic proteins dampen inflammasome pathway activation by stabilizing mitochondria and/or maintaining mitochondrial quality control through autophagy.

In contrast to negative regulation of autophagy by the inflammasome, a recent study demonstrates that autophagy induction by starvation enhances caspase-1 activation and secretion of IL-1*β* and IL-18 [126]. Inflammasome-mediated IL-1*β* secretion utilizes the autophagy-based unconventional secretion pathway [126]. It is possible that a distinct type of autophagy induction might differentially regulate the inflammasome pathway.

Taken together these studies suggest an important role for autophagic proteins in the dampening of proinflammatory responses, which warrants further investigation in models of inflammatory disease.

In addition to confirmed negative regulatory roles of autophagy in inflammasome activation, it has been shown that stimulation of inflammasome pathways can promote autophagosome formation through activation of the GTPase RalB [127]. Furthermore, p62-dependent selective autophagy processes may regulate the turnover and degradation of ubiquitinated inflammasome complexes [127]. Further studies suggest that stimulation of plasminogen activator inhibitor-2 by Toll like receptor activation suppresses NLRP3-dependent cytokines activation by promoting the autophagic degradation of NLRP3 [128]. An important and unanswered question is related to whether inflammasome activation and the generation of inflammasome-associated cytokines exert downstream consequences on autophagic processing.

Pyroptosis is triggered in inflammatory cells in response to excessive inflammation by caspase-1-dependent processes, leading to release of proinflammatory cytokines (e.g., IL-1*β*, IL-18, and IL-33) from dying cells. Recent studies suggest that macrophages activate autophagy in parallel with inflammasome activation, as a means to delay the onset of pyroptosis [129]. Chemical inhibition of autophagy using 3-methyladenine or inhibition of the Atg4 protease resulted in increased incidence of pyroptotic cell death in activated macrophages [129]. The impact of autophagy modulation on the regulation of pyroptosis, and the relevance of these interactions in *in vivo* models of inflammatory disease and sepsis, warrants further exploration.

## 6. Conclusions and Therapeutic Implications

**Figure 3:**
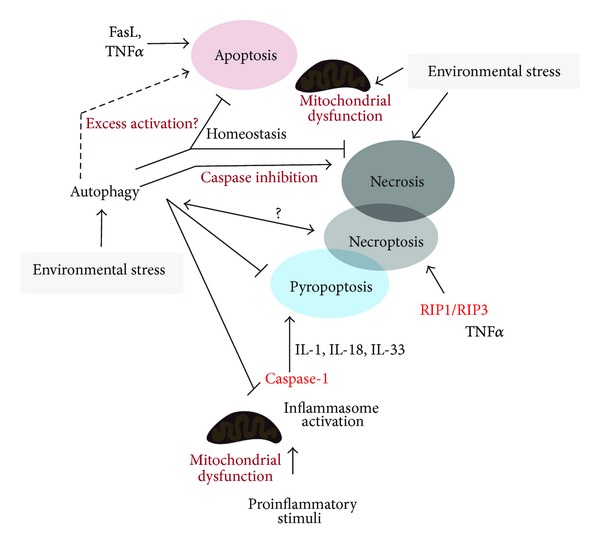
Autophagy has a complex relationship with various modes of cell death, including regulated (e.g., apoptosis, pyroptosis, and necroptosis) and catastrophic (e.g., necrosis) types of cell death. Autophagy has been implicated in association with caspase-independent cell death in apoptosis-compromised cells leading to necrosis and necroptosis. Furthermore, autophagy has been implicated as an inhibitor of both apoptosis and necrosis by preserving cellular functions, removing toxic debris, and maintaining cellular energy charge. Nevertheless, proapoptotic roles of autophagy have also been reported. Proinflammatory stimuli can activate inflammasome-dependent caspase-1 activation leading to proinflammatory cytokines maturation. Excess activation of this pathway can lead to pyroptotic cell death. Mitochondrial dysfunction plays a key role in both apoptosis signaling and the activation of the inflammasome pathway. Autophagy can influence these pathways through modulation of the mitochondrial pool. The relationships between autophagy and necroptosis or pyroptosis require further elucidation.

Autophagy is generally defined as a cellular program that ensures survival under conditions of stress. The ability of autophagy to clear damaged or denatured subcellular constituents such as aggregated protein (i.e., aggrephagy) as well as to maintain mitochondrial homeostasis (i.e., mitophagy) appears to play important roles in the cytoprotective and homeostatic functions of autophagy [20]. Despite the homeostatic roles, autophagy is now recognized to play complex and incompletely understood roles in cell death programs (Figure 3). Furthermore, there is considerable cross-talk between the molecular regulation of autophagy and other regulated forms of cell death [23–26]. The role of autophagy in diseases is an emerging area of investigation, with recent studies indicating that autophagy may exert multifunctional roles in specific diseases, with the potential for both adaptive and harmful outcomes. Furthermore, deficiency or absence in autophagic function may play a pathogenic role in select human diseases [2, 17–19]. Additional studies are needed to define the dynamic equilibrium between autophagy, apoptosis, regulated necrosis, and other modes of cell death in the context of human disease pathogenesis [121]. Furthermore, additional studies are needed to determine the relevance of autophagic regulation of pyroptosis in inflammatory diseases [130]. An increased understanding of these relationships would be essential in the development of therapeutics targeting the autophagy pathway for the treatment of disease.

## References

[1] Levine B, Klionsky DJ (2004). Development by self-digestion: molecular mechanisms and biological functions of autophagy. *Developmental Cell*.

[2] Mizushima N, Levine B, Cuervo AM, Klionsky DJ (2008). Autophagy fights disease through cellular self-digestion. *Nature*.

[3] Klionsky DJ, Emr SD (2000). Autophagy as a regulated pathway of cellular degradation. *Science*.

[4] Eskelinen EL, Saftig P (2009). Autophagy: a lysosomal degradation pathway with a central role in health and disease. *Biochimica et Biophysica Acta*.

[5] Ravikumar B, Sarkar S, Davies JE (2010). Regulation of mammalian autophagy in physiology and pathophysiology. *Physiological Reviews*.

[6] Kelekar A (2005). Autophagy. *Annals of the New York Academy of Sciences*.

[7] Johansen T, Lamark T (2011). Selective autophagy mediated by autophagic adapter proteins. *Autophagy*.

[8] Lamark T, Johansen T (2012). Aggrephagy: selective disposal of protein aggregates by macroautophagy. *International Journal of Cell Biology*.

[9] Youle RJ, Narendra DP (2011). Mechanisms of mitophagy. *Nature Reviews Molecular Cell Biology*.

[10] Sumpter R, Levine B (2010). Autophagy and innate immunity: triggering, targeting and tuning. *Seminars in Cell and Developmental Biology*.

[11] Kroemer G, Mariño G, Levine B (2010). Autophagy and the integrated stress response. *Molecular Cell*.

[12] Nakahira K, Cloonan SM, Mizumura K, Choi AM, Ryter SW (2013). Autophagy: a crucial moderator of redox balance, inflammation, and apoptosis in lung disease. *Antioxidants and Redox Signalling*.

[13] Ryter SW, Cloonan SM, Choi AM (2013). Autophagy: a critical regulator of cellular metabolism and homeostasis. *Molecules and Cells*.

[14] Ryter SW, Choi AM (2013). Regulation of autophagy in oxygen-dependent cellular stress. *Current Pharmaceutical Design*.

[15] Levine B, Mizushima N, Virgin HW (2011). Autophagy in immunity and inflammation. *Nature*.

[16] Deretic V, Levine B (2009). Autophagy, immunity, and microbial adaptations. *Cell Host and Microbe*.

[17] Choi AM, Ryter SW, Levine B (2013). Autophagy in human health and disease. *New England Journal of Medicine*.

[18] Rubinsztein DC, Codogno P, Levine B (2012). Autophagy modulation as a potential therapeutic target for diverse diseases. *Nature Reviews Drug Discovery*.

[19] Levine B, Kroemer G (2008). Autophagy in the pathogenesis of disease. *Cell*.

[20] Mizushima N, Komatsu M (2011). Autophagy: renovation of cells and tissues. *Cell*.

[21] Kuma A, Hatano M, Matsui M (2004). The role of autophagy during the early neonatal starvation period. *Nature*.

[22] Boya P, González-Polo R, Casares N (2005). Inhibition of macroautophagy triggers apoptosis. *Molecular and Cellular Biology*.

[23] Galluzzi L, Vicencio JM, Kepp O, Tasdemir E, Maiuri MC, Kroemer G (2008). To die or not to die: that is the autophagic question. *Current Molecular Medicine*.

[24] Maiuri MC, Zalckvar E, Kimchi A, Kroemer G (2007). Self-eating and self-killing: crosstalk between autophagy and apoptosis. *Nature Reviews Molecular Cell Biology*.

[25] Kroemer G, Levine B (2008). Autophagic cell death: the story of a misnomer. *Nature Reviews Molecular Cell Biology*.

[26] Shen HM, Codogno P (2011). Autophagic cell death: Loch Ness monster or endangered species?. *Autophagy*.

[27] Shimizu S, Kanaseki T, Mizushima N (2004). Role of Bcl-2 family proteins in a non-apoptopic programmed cell death dependent on autophagy genes. *Nature Cell Biology*.

[28] Jain MV, Paczulla AM, Klonisch T (2013). Interconnections between apoptotic, autophagic and necrotic pathways: implications for cancer therapy development. *Journal of Cellular and Molecular Medicine*.

[29] Su M, Mei Y, Sinha S (2013). Role of the crosstalk between autophagy and apoptosis in cancer. *Journal of Oncology*.

[30] Galluzzi L, Maiuri MC, Vitale I (2007). Cell death modalities: classification and pathophysiological implications. *Cell Death and Differentiation*.

[31] Kroemer G, Galluzzi L, Vandenabeele P (2009). Classification of cell death: recommendations of the nomenclature committee on cell death 2009. *Cell Death and Differentiation*.

[32] Fink SL, Cookson BT (2005). Apoptosis, pyroptosis, and necrosis: mechanistic description of dead and dying eukaryotic cells. *Infection and Immunity*.

[33] Majno G, Joris I (1995). Apoptosis, oncosis, and necrosis: an overview of cell death. *American Journal of Pathology*.

[34] Galluzzi L, Kroemer G (2008). Necroptosis: a specialized pathway of programmed necrosis. *Cell*.

[35] Vandenabeele P, Galluzzi L, Vanden Berghe T, Kroemer G (2010). Molecular mechanisms of necroptosis: an ordered cellular explosion. *Nature Reviews Molecular Cell Biology*.

[36] Zhang DW, Shao J, Lin J (2009). RIP3, an energy metabolism regulator that switches TNF-induced cell death from apoptosis to necrosis. *Science*.

[37] He S, Wang L, Miao L (2009). Receptor interacting protein kinase-3 determines cellular necrotic response to TNF-*α*. *Cell*.

[38] Cho YS, Challa S, Moquin D (2009). Phosphorylation-driven assembly of the RIP1-RIP3 complex regulates programmed necrosis and virus-induced inflammation. *Cell*.

[39] Galluzzi L, Vanden Berghe T, Vanlangenakker N (2011). Programmed necrosis. From molecules to health and disease. *International Review of Cell and Molecular Biology*.

[40] Sun L, Wang H, Wang Z (2012). Mixed lineage kinase domain-like protein mediates necrosis signaling downstream of RIP3 kinase. *Cell*.

[41] Welz PS, Wullaert A, Vlantis K (2011). FADD prevents RIP3-mediated epithelial cell necrosis and chronic intestinal inflammation. *Nature*.

[42] Günther C, Martini E, Wittkopf N (2011). Caspase-8 regulates TNF-*α*-induced epithelial necroptosis and terminal ileitis. *Nature*.

[43] Kepp O, Galluzzi L, Zitvogel L, Kroemer G (2010). Pyroptosis-a cell death modality of its kind?. *European Journal of Immunology*.

[44] Cookson BT, Brennan MA (2001). Pro-inflammatory programmed cell death. *Trends in Microbiology*.

[45] Yang Z, Klionsky DJ (2010). Mammalian autophagy: core molecular machinery and signaling regulation. *Current Opinion in Cell Biology*.

[46] He C, Klionsky DJ (2009). Regulation mechanisms and signaling pathways of autophagy. *Annual Review of Genetics*.

[47] Jung CH, Ro S, Cao J, Otto NM, Kim Do-Hyung D-H (2010). MTOR regulation of autophagy. *FEBS Letters*.

[48] Mizushima N (2010). The role of the Atg1/ULK1 complex in autophagy regulation. *Current Opinion in Cell Biology*.

[49] Chan EY (2012). Regulation and function of uncoordinated-51 like kinase proteins. *Antioxidants & Redox Signaling*.

[50] Hosokawa N, Hara T, Kaizuka T (2009). Nutrient-dependent mTORCl association with the ULK1-Atg13-FIP200 complex required for autophagy. *Molecular Biology of the Cell*.

[51] Ganley IG, Lam DH, Wang J, Ding X, Chen S, Jiang X (2009). ULK1.ATG13.FIP200 complex mediates mTOR signaling and is essential for autophagy. *Journal of Biological Chemistry*.

[52] Jung CH, Jun CB, Ro S (2009). ULK-Atg13-FIP200 complexes mediate mTOR signaling to the autophagy machinery. *Molecular Biology of the Cell*.

[53] Wong PM, Puente C, Ganley IG, Jiang X (2013). The ULK1 complex: sensing nutrient signals for autophagy activation. *Autophagy*.

[54] Hara T, Takamura A, Kishi C (2008). FIP200, a ULK-interacting protein, is required for autophagosome formation in mammalian cells. *Journal of Cell Biology*.

[55] Mihaylova MM, Shaw RJ (2011). The AMPK signalling pathway coordinates cell growth, autophagy and metabolism. *Nature Cell Biology*.

[56] Kim J, Kundu M, Viollet B, Guan K (2011). AMPK and mTOR regulate autophagy through direct phosphorylation of Ulk1. *Nature Cell Biology*.

[57] He C, Levine B (2010). The Beclin 1 interactome. *Current Opinion in Cell Biology*.

[58] Itakura E, Kishi C, Inoue K, Mizushima N (2008). Beclin 1 forms two distinct phosphatidylinositol 3-kinase complexes with mammalian Atg14 and UVRAG. *Molecular Biology of the Cell*.

[59] Wang RC, Wei Y, An Z (2012). Akt-mediated regulation of autophagy and tumorigenesis through Beclin 1 phosphorylation. *Science*.

[60] Pattingre S, Tassa A, Qu X (2005). Bcl-2 antiapoptotic proteins inhibit Beclin 1-dependent autophagy. *Cell*.

[61] Ohsumi Y (2001). Molecular dissection of autophagy: two ubiquitin-like systems. *Nature Reviews Molecular Cell Biology*.

[62] Tanida I, Ueno T, Kominami E (2004). LC3 conjugation system in mammalian autophagy. *International Journal of Biochemistry and Cell Biology*.

[63] Kabeya Y, Mizushima N, Ueno T (2000). LC3, a mammalian homologue of yeast Apg8p, is localized in autophagosome membranes after processing. *EMBO Journal*.

[64] Kabeya Y, Mizushima N, Yamamoto A, Oshitani-Okamoto S, Ohsumi Y, Yoshimori T (2004). LC3, GABARAP and GATE16 localize to autophagosomal membrane depending on form-II formation. *Journal of Cell Science*.

[65] He H, Dang Y, Dai F (2003). Post-translational modifications of three members of the human MAP1LC3 family and detection of a novel type of modification for MAP1LC3B. *Journal of Biological Chemistry*.

[66] Satoo K, Noda NN, Kumeta H (2009). The structure of Atg4B-LC3 complex reveals the mechanism of LC3 processing and delipidation during autophagy. *EMBO Journal*.

[67] Zhang H, Bosch-Marce M, Shimoda LA (2008). Mitochondrial autophagy is an HIF-1-dependent adaptive metabolic response to hypoxia. *Journal of Biological Chemistry*.

[68] Ding WX, Ni HM, Li M (2010). Nix is critical to two distinct phases of mitophagy, reactive oxygen species-mediated autophagy induction and Parkin-ubiquitin-p62-mediated mitochondrial priming. *Journal of Biological Chemistry*.

[69] Narendra D, Tanaka A, Suen D, Youle RJ (2008). Parkin is recruited selectively to impaired mitochondria and promotes their autophagy. *Journal of Cell Biology*.

[70] Geisler S, Holmström KM, Skujat D (2010). PINK1/Parkin-mediated mitophagy is dependent on VDAC1 and p62/SQSTM1. *Nature Cell Biology*.

[71] Morris HR (2005). Genetics of Parkinson’s disease. *Annals of Medicine*.

[72] Trancikova A, Tsika E, Moore DJ (2012). Mitochondrial dysfunction in genetic animal models of Parkinson’s disease. *Antioxidants and Redox Signaling*.

[73] Vives-Bauza C, Zhou C, Huang Y (2010). PINK1-dependent recruitment of Parkin to mitochondria in mitophagy. *Proceedings of the National Academy of Sciences of the United States of America*.

[74] Chen Y, Dorn GW (2013). PINK1-phosphorylated mitofusin 2 is a Parkin receptor for culling damaged mitochondria. *Science*.

[75] Sarraf SA, Raman M, Guarani-Pereira V (2013). Landscape of the PARKIN-dependent ubiquitylome in response to mitochondrial depolarization. *Nature*.

[76] Ichimura Y, Komatsu M (2010). Selective degradation of p62 by autophagy. *Seminars in Immunopathology*.

[77] Shaid S, Brandts CH, Serve H, Dikic I (2013). Ubiquitination and selective autophagy. *Cell Death Differentiation*.

[78] Kirkin V, Lamark T, Sou Y (2009). A role for NBR1 in autophagosomal degradation of ubiquitinated substrates. *Molecular Cell*.

[79] Clausen TH, Lamark T, Isakson P (2010). p62/SQSTM1 and ALFY interact to facilitate the formation of p62 bodies/ALIS and their degradation by autophagy. *Autophagy*.

[80] Deosaran E, Larsen KB, Hua R (2013). NBR1 acts as an autophagy receptor for peroxisomes. *Journal of Cell Science*.

[81] MacIntosh GC, Bassham DC (2011). The connection between ribophagy, autophagy and ribosomal RNA decay. *Autophagy*.

[82] Cebollero E, Reggiori F, Kraft C (2012). Reticulophagy and ribophagy: regulated degradation of protein production factories. *International Journal of Cell Biology*.

[83] Liu K, Czaja MJ (2013). Regulation of lipid stores and metabolism by lipophagy. *Cell Death and Differentiation*.

[84] Fujiwara Y, Furuta A, Kikuchi H (2013). Discovery of a novel type of autophagy targeting RNA. *Autophagy*.

[85] Robert G, Gastaldi C, Puissant A (2012). The anti-apoptotic Bcl-B protein inhibits BECN1-dependent autophagic cell death. *Autophagy*.

[86] Zhu Y, Massen S, Terenzio M (2013). Modulation of serines 17 and 24 in the LC3-interacting region of Bnip3 determines pro-survival mitophagy versus apoptosis. *Journal of Biological Chemistry*.

[87] Luo S, Rubinsztein DC (2010). Apoptosis blocks Beclin 1-dependent autophagosome synthesis: an effect rescued by Bcl-xL. *Cell Death and Differentiation*.

[88] Pyo JO, Jang M, Kwon Y (2005). Essential roles of Atg5 and FADD in autophagic cell death: dissection of autophagic cell death into vacuole formation and cell death. *Journal of Biological Chemistry*.

[89] Yousefi S, Perozzo R, Schmid I (2006). Calpain-mediated cleavage of Atg5 switches autophagy to apoptosis. *Nature Cell Biology*.

[90] Rubinstein AD, Eisenstein M, Ber Y, Bialik S, Kimchi A (2011). The autophagy protein atg12 associates with antiapoptotic Bcl-2 family members to promote mitochondrial apoptosis. *Molecular Cell*.

[91] Betin VM, Lane JD (2009). Caspase cleavage of Atg4D stimulates GABARAP-L1 processing and triggers mitochondrial targeting and apoptosis. *Journal of Cell Science*.

[92] Oral O, Oz-Arslan D, Itah Z (2012). Cleavage of Atg3 protein by caspase-8 regulates autophagy during receptor-activated cell death. *Apoptosis*.

[93] Lee JS, Li Q, Lee J (2009). FLIP-mediated autophagy regulation in cell death control. *Nature Cell Biology*.

[94] Crighton D, Wilkinson S, O’Prey J (2006). DRAM, a p53-induced modulator of autophagy, is critical for apoptosis. *Cell*.

[95] Feng Z, Zhang H, Levine AJ, Jin S (2005). The coordinate regulation of the p53 and mTOR pathways in cells. *Proceedings of the National Academy of Sciences of the United States of America*.

[96] Tasdemir E, Maiuri MC, Galluzzi L (2008). Regulation of autophagy by cytoplasmic p53. *Nature Cell Biology*.

[97] Contreras AU, Mebratu Y, Delgado M (2013). Deacetylation of p53 induces autophagy by suppressing Bmf expression. *Journal of Cell Biology*.

[98] Tasdemir E, Maiuri MC, Morselli E (2008). A dual role of p53 in the control of autophagy. *Autophagy*.

[99] Levine B, Abrams J (2008). p53: the Janus of autophagy?. *Nature Cell Biology*.

[100] Chen ZH, Kim HP, Sciurba FC (2008). Egr-1 regulates autophagy in cigarette smoke-induced chronic obstructive pulmonary disease. *PLoS ONE*.

[101] Chen ZH, Lam HC, Jin Y (2010). Autophagy protein microtubule-associated protein 1 light chain-3B (LC3B) activates extrinsic apoptosis during cigarette smoke-induced emphysema. *Proceedings of the National Academy of Sciences of the United States of America*.

[102] Cha HH, Hwang JR, Kim HY, Choi SJ, Oh SY, Roh CR (2013). Autophagy induced by tumor necrosis factor *α* mediates intrinsic apoptosis in trophoblastic cells. *Reproductive Sciences*.

[103] Wang Y, Singh R, Massey AC (2008). Loss of macroautophagy promotes or prevents fibroblast apoptosis depending on the death stimulus. *Journal of Biological Chemistry*.

[104] Xu Y, Sung OK, Li Y, Han J (2006). Autophagy contributes to caspase-independent macrophage cell death. *Journal of Biological Chemistry*.

[105] Yu L, Wan F, Dutta S (2006). Autophagic programmed cell death by selective catalase degradation. *Proceedings of the National Academy of Sciences of the United States of America*.

[106] Madden DT, Egger L, Bredesen DE (2007). A calpain-like protease inhibits autophagic cell death. *Autophagy*.

[107] Yu L, Alva A, Su H (2004). Regulation of an ATG7-beclin 1 program of autophaglic cell death by caspase-8. *Science*.

[108] Wu YT, Tan HL, Huang Q (2008). Autophagy plays a protective role during zVAD-induced necrotic cell death. *Autophagy*.

[109] Shimizu S, Kanaseki T, Mizushima N (2004). Role of Bcl-2 family proteins in a non-apoptopic programmed cell death dependent on autophagy genes. *Nature Cell Biology*.

[110] Mathew R, Karantza-Wadsworth V, White E (2007). Role of autophagy in cancer. *Nature Reviews Cancer*.

[111] Degenhardt K, Mathew R, Beaudoin B (2006). Autophagy promotes tumor cell survival and restricts necrosis, inflammation, and tumorigenesis. *Cancer Cell*.

[112] Jin S, DiPaola RS, Mathew R, White E (2007). Metabolic catastrophe as a means to cancer cell death. *Journal of Cell Science*.

[113] Lemasters JJ, Nieminen A, Qian T (1998). The mitochondrial permeability transition in cell death: a common mechanism in necrosis, apoptosis and autophagy. *Biochimica et Biophysica Acta*.

[114] Loos B, Engelbrecht AM, Lockshin RA, Klionsky DJ, Zakeri Z (2013). The variability of autophagy and cell death susceptibility: unanswered questions. *Autophagy*.

[115] Eisenberg T, Knauer H, Schauer A (2009). Induction of autophagy by spermidine promotes longevity. *Nature Cell Biology*.

[116] Bonapace L, Bornhauser BC, Schmitz M (2010). Induction of autophagy-dependent necroptosis is required for childhood acute lymphoblastic leukemia cells to overcome glucocorticoid resistance. *Journal of Clinical Investigation*.

[117] Bell BD, Leverrier S, Weist BM (2008). FADD and caspase-8 control the outcome of autophagic signaling in proliferating T cells. *Proceedings of the National Academy of Sciences of the United States of America*.

[118] Khan MJ, Rizwan Alam M, Waldeck-Weiermair M (2012). Inhibition of autophagy rescues palmitic acid-induced necroptosis of endothelial cells. *Journal of Biological Chemistry*.

[119] Wang YQ, Wang L, Zhang MY (2012). Necrostatin-1 suppresses autophagy and apoptosis in mice traumatic brain injury model. *Neurochemical Research*.

[120] Lu JV, Walsh CM (2012). Programmed necrosis and autophagy in immune function. *Immunology Reviews*.

[121] Nikoletopoulou V, Markaki M, Palikaras K, Tavernarakis N (2013). Crosstalk between apoptosis, necrosis and autophagy. *Biochimica Biophysica Acta*.

[122] Saitoh T, Fujita N, Jang MH (2008). Loss of the autophagy protein Atg16L1 enhances endotoxin-induced IL-1*β* production. *Nature*.

[123] Nakahira K, Haspel JA, Rathinam VAK (2011). Autophagy proteins regulate innate immune responses by inhibiting the release of mitochondrial DNA mediated by the NALP3 inflammasome. *Nature Immunology*.

[124] Zhou R, Yazdi AS, Menu P, Tschopp J (2011). A role for mitochondria in NLRP3 inflammasome activation. *Nature*.

[125] Schroder K, Tschopp J (2010). The Inflammasomes. *Cell*.

[126] Dupont N, Jiang S, Pilli M, Ornatowski W, Bhattacharya D, Deretic V (2011). Autophagy-based unconventional secretory pathway for extracellular delivery of IL-1*β*. *EMBO Journal*.

[127] Shi CS, Shenderov K, Huang NN (2012). Activation of autophagy by inflammatory signals limits IL-1*β* production by targeting ubiquitinated inflammasomes for destruction. *Nature Immunology*.

[128] Chuang SY, Yang CH, Chou CC, Chiang YP, Chuang TH, Hsu LC (2013). TLR-induced PAI-2 expression suppresses IL-1*β* processing via increasing autophagy and NLRP3 degradation. *Proceedings of the National Academy of Sciences of the United States of America*.

[129] Byrne BG, Dubuisson JF, Joshi AD, Persson JJ, Swanson MS (2013). Inflammasome components coordinate autophagy and pyroptosis as macrophage responses to infection. *MBio*.

[130] Bortoluci KR, Medzhitov R (2010). Control of infection by pyroptosis and autophagy: role of TLR and NLR. *Cellular and Molecular Life Sciences*.

